# One Ring to Rule Them All and in the Darkness Bind Them: A Report of Two Cases of Penile Strangulation and Timely Intervention

**DOI:** 10.7759/cureus.76931

**Published:** 2025-01-05

**Authors:** Devesh Dhamor, Kapil Bajaj, Aditya P Sharma

**Affiliations:** 1 Urology, Postgraduate Institute of Medical Education and Research (PGIMER), Chandigarh, IND

**Keywords:** penile strangulation, sexual disorders, sexual emergency, sexual paraphilia, sexual risk behaviors

## Abstract

Penile strangulation is a rare but serious urological emergency requiring prompt intervention to prevent severe complications such as ischemia and necrosis. Typically self-inflicted, the condition arises when a constricting object obstructs venous and lymphatic outflow, leading to progressive edema. Metallic rings, often implicated in such cases, pose unique challenges as they become lodged behind the edematous tissue, rendering standard hospital equipment ineffective for removal. Management requires a tailored approach, often utilizing specialized tools like metal cutters. Here, we discuss the presentation and management of two such cases treated at our institution.

## Introduction

Penile strangulation is a rare condition but is a potentially serious urological emergency. The foreign body encircling the penis obstructs the venous and lymphatic outflow [1]. Such actions are usually self-inflicted to enhance sexual activity by maintaining prolonged erections. However, the resultant edema can inhibit the simple removal of the encircling object, leading to ischemia and necrosis if not promptly addressed [2]. There exists no standard of care for management in such presentations. Moreover, these metal rings get stuck behind the edematous phallus and cannot be removed by usual maneuvers. Most of the standard hospital equipment available is not of much use in such situations, and handyman tools are often required for removal. Special metal cutters are needed to cut and remove these rings. Each case needs to be managed individually based on the object involved and the severity of the underlying injury. Here, we present two cases of penile strangulation with foreign objects that presented to the surgical emergency at our institution and their management.

## Case presentation

Case 1

**Figure 1:**
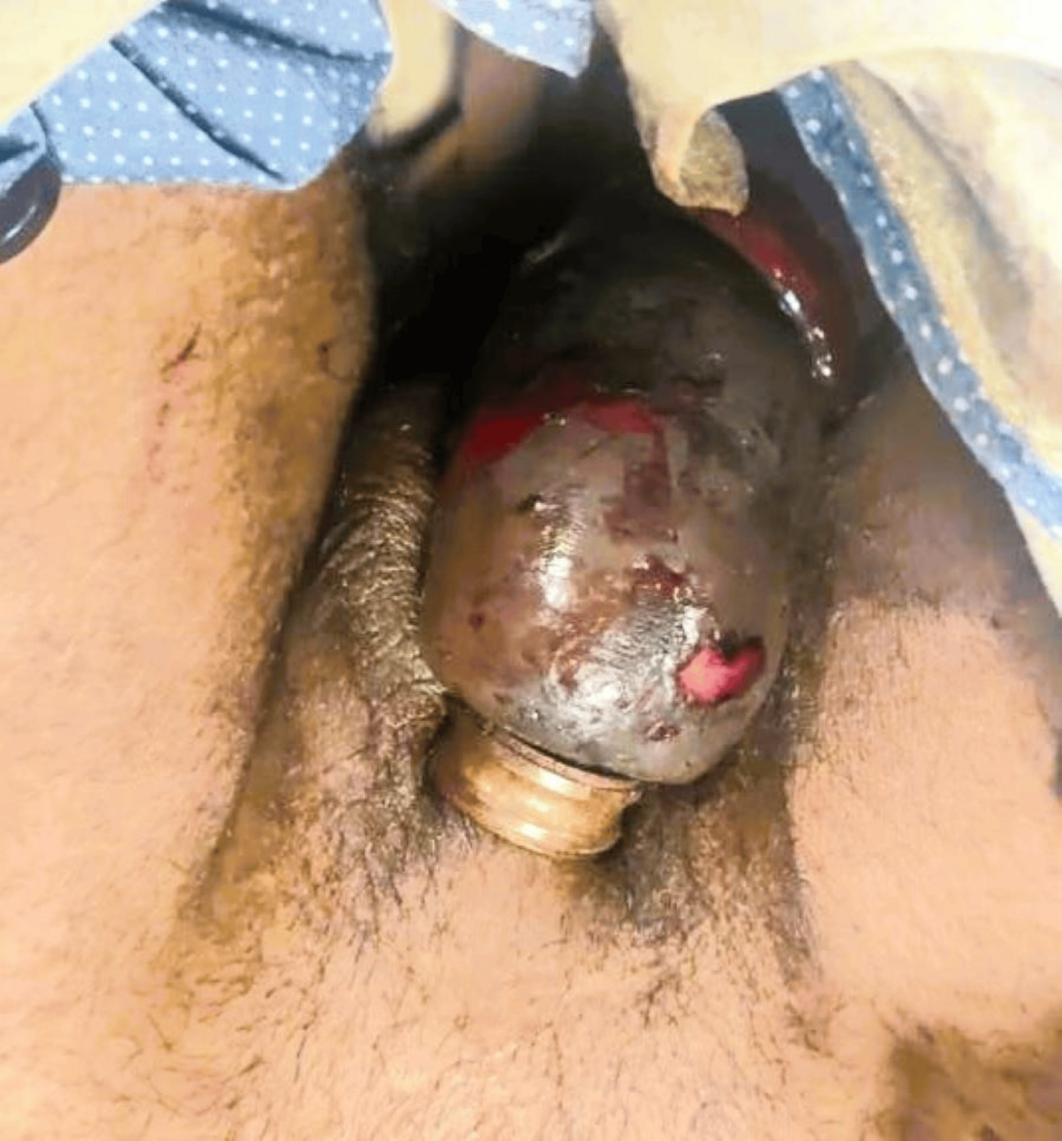
Strangulating foreign body around the proximal shaft

A 30-year-old man presented to the emergency department with a three-day history of penile swelling caused by a metal bearing encircling the proximal penile shaft resulting from the purpose of self-enhancing erectile duration. Examination revealed marked edema, congestion, and areas of skin desquamation with non-viable tissue demarcated at the level of constriction (Figure 1). The blood workup was grossly normal. A psychiatric evaluation was done which was not suggestive of any underlying psychiatric disorder. Using an electric saw, the metal bearing was carefully cut and removed. The necrotic tissue was subsequently debrided. The urethra was found to be intact, allowing for the smooth passage of a per-urethral catheter. The patient was managed with serial hygroscopic dressings and showed significant recovery, with resolution of edema and congestion, leading to discharge in stable condition after five days of admission. The patient was followed up till one month after discharge and thereafter lost to follow-up.

Case 2

**Figure 2:**
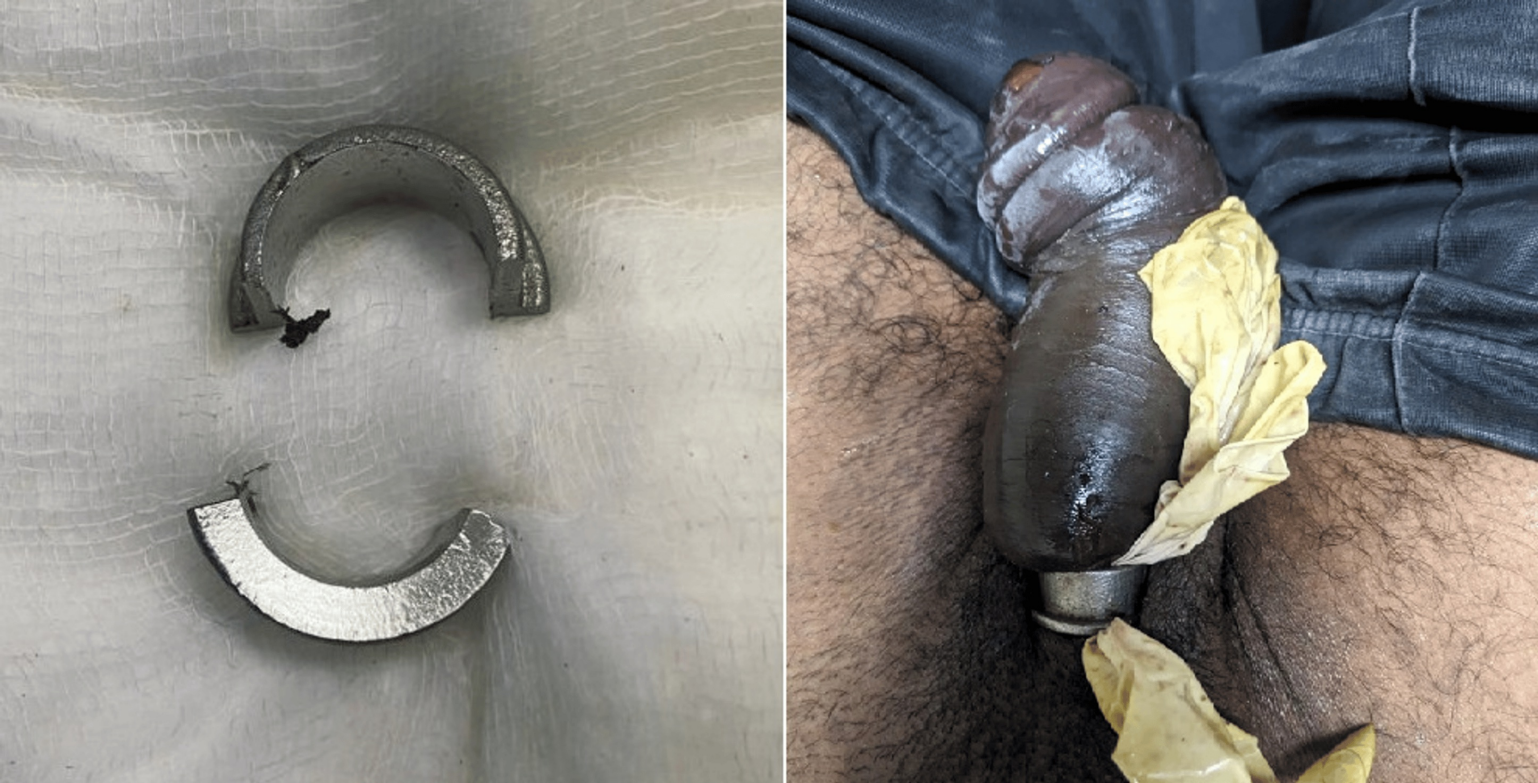
Right: Constricting ring placed around the proximal penile shaft with distal edema and desquamation. Left: Cut open constricting ring

A 42-year-old man presented to the surgical emergency with a 20-hour history of placement of a constriction band (metal ring) over his penis to enhance sexual activity. At presentation, he had pain in his penis; on examination, his penis was edematous with areas of skin desquamation, without any region of frank gangrene (Figure 2). An attempt was made to remove the ring by greasing and milking the ring out which, however, failed. A wire cutter was used to cut the ring on two ends to relieve the compression. His laboratory investigations were normal, and psychiatric evaluation was conclusive in ruling out any psychiatric abnormality. There were no signs of urethral injury, and a per-urethral catheter could easily be passed into the bladder. He was followed up serially with hygroscopic dressings and discharged after two days with resolved penile edema. He has been followed up till one-year post-discharge and has no erectile dysfunction at present.

## Discussion

While penile strangulation is uncommon, it is reported across various age groups, from adolescents to older adults, with most cases being adolescents or young adults. The motivation behind such practices varies, including sexual experimentation, self-treatment of erectile dysfunction, psychiatric conditions, or sometimes colleague antics [3].

Outflow obstruction by using rings may initially result in a persistent erection, a property utilized in vacuum erection devices (VED) for the treatment of erectile dysfunction. However, it subsequently results in venous and lymphatic occlusion-mediated edema, preventing the safe removal of the constriction band. If not addressed in time, the progressing edema results in compromised arterial inflow to the penis, which eventually leads to ischemia and necrosis [1].

Such rings may be metallic or non-metallic in nature. Non-metallic rings usually do not present in hospital settings since they are easily addressed with simple cutting tools. Most patients presenting to healthcare settings present with metallic rings with a prolonged insult history.

Patients may present at varied stages of strangulation. Bhat et al. categorized penile strangulation injuries into five grades ranging from edema of the penis (grade 1) to gangrene and necrosis of the penis (grade 5) [4].

The management of such patients is not standardized. The treating team must be creative and adaptive due to the varied nature of constricting objects to tackle the situation in the most rapid and safest method possible. Most of the instruments used are potent in causing harm, and one must be careful to only cut the object and not let it injure the underlying tissue. Other than the risk of direct tissue injury, there is the additional risk of thermal injury associated with the grazing of metal against metal while cutting the constricting body. Various practitioners have described their own innovative methods for the same ranging from the use of basic handyman tools like mechanical or electric saws, wire cutters, or drills to the use of orthopedic or dental equipment like Gigli saw, bone saw, or Lister scissors [1,5-7]. Some methods including greasing and milking out the constriction band are successful only when penile edema is not significant and often have already been attempted by patients themselves before presentation to a healthcare setting.

Along with the addressal of the noxious agent, the associated injury to the underlying tissue needs to be thoroughly evaluated and addressed. It is imperative to debride any devitalized tissue which may subsequently get infected. It is also important to rule out urethral injury and address the same with initial diversion or repair depending on the viability of surrounding tissues. The above may entail minimal excision of devitalized tissue as in our case along with closure, diversion in the form of a suprapubic catheter, or even a total penectomy depending upon the severity of tissue damage [4]. 

The long-term outcomes associated with these injuries are dependent on the rapidity of treatment. The outcomes, if the obstruction is relieved when gangrene has not set in, are excellent, with complete recovery of pre-insult erectile function within a few days. As the time to treatment increases, so does the underlying ischemia and radicality of treatment. In the long term, this may result in the disfigurement of the penis, erectile dysfunction owing to injury to the arterial inflow, tunica albuginea or cavernosal fibrosis, urethral stricture and/or fistula formation, etc. [1].

## Conclusions

Penile strangulation with a foreign body is a time-sensitive condition requiring prompt management. The presentation time since the injury is of utmost importance with early presentations having no long-term complications. It is also necessary to rule out any underlying psychiatric illness and its addressal before discharge of such patients to prevent recurrent episodes. Awareness among healthcare providers is essential for early diagnosis, prompt referral, effective treatment, and prevention of complications.
